# Trajectories of Health-Related Quality of Life in Patients With Idiopathic Pulmonary Fibrosis

**DOI:** 10.1016/j.chpulm.2024.100133

**Published:** 2024-12-27

**Authors:** Megan L. Neely, Jamie L. Todd, Laurie D. Snyder, Peide Li, Amy L. Olson, IPF-PRO Registry Investigators

**Affiliations:** a Duke Clinical Research Institute, Durham, NC; b Duke University Medical Center, Durham, NC; c Boehringer Ingelheim Pharmaceuticals, Inc, Ridgefield, CT

**Keywords:** interstitial lung disease, patient-reported outcome measures, pulmonary fibrosis

## Abstract

**Background:**

Patients with idiopathic pulmonary fibrosis (IPF) experience impairments in health-related quality of life (HRQL).

**Research Question:**

What is the trajectory of decline in HRQL in patients with IPF and is this influenced by patients’ demographic/clinical characteristics at baseline?

**Study Design and Methods:**

The Idiopathic Pulmonary Fibrosis Prospective Outcomes (IPF-PRO) Registry is a registry of patients with IPF. HRQL was assessed at enrollment and during routine clinical care using the following patient-reported outcomes (PROs): the Cough and Sputum Assessment Questionnaire, the St. George’s Respiratory Questionnaire, the 12-item Short Form Survey, and the EuroQol score and visual analog scale. Trajectories of PRO scores were estimated using a mixed joint model. Associations between sex, age, FVC and diffusing capacity of the lungs for carbon monoxide (both % predicted), use of supplemental oxygen, and use of antifibrotic therapy at enrollment and trajectories of PRO scores were assessed.

**Results:**

The cohort included 957 patients. Estimated mean changes in scores over 48 months were 10.8, 7.0, 13.1, and 10.5 for the St. George’s Respiratory Questionnaire total, symptoms, activity, and impact scores; −7.6 and −6.5 for the Cough and Sputum Assessment Questionnaire cough impact and symptoms scores; −2.1 and −7.0 for the 12-item Short Form Survey Mental Component Summary and Physical Component Summary scores; and −0.08 and −9.0 for the EuroQol score and visual analog scale, respectively (*P* < .001 for all). At enrollment, female sex, lower age, lower FVC and diffusing capacity of the lungs for carbon monoxide % predicted, and use of supplemental oxygen were associated with worse PRO scores. Trajectories of decline in PRO scores were similar across levels of demographic/clinical factors assessed at enrollment.

**Interpretation:**

Among patients in the IPF-PRO Registry, a range of PROs showed worsening in HRQL over 48 months. Female sex, lower age, worse lung function, and use of supplemental oxygen were associated with worse HRQL at enrollment and during follow-up. These findings suggest that interventions aimed at preserving HRQL in patients with IPF may be of particular benefit for certain groups of patients.

**Clinical Trial Registration:**

ClinicalTrials.gov; No.: NCT01915511; URL: www.clinicaltrials.gov

**Take-Home Points**

**Study Question:** What is the trajectory of decline in health-related quality of life (HRQL) in patients with idiopathic pulmonary fibrosis (IPF) and is this influenced by patients’ demographic/clinical characteristics?

**Results:** Scores on several patient-reported outcomes worsened over 48 months, with lower age, female sex, worse lung function, and use of supplemental oxygen associated with worse HRQL at baseline and during follow-up.

**Interpretation:** Interventions aimed at preserving HRQL in patients with IPF are needed and may be of particular benefit for certain groups of patients.

Idiopathic pulmonary fibrosis (IPF) is a fibrosing interstitial lung disease (ILD) associated with progressive lung function decline and premature death.^1^ IPF is associated with impaired health-related quality of life (HRQL), particularly related to symptoms of cough, dyspnea, and fatigue and loss of physical function,^2^^,^^3^ which worsens as the disease progresses.^4^, ^5^, ^6^ A variety of patient-reported outcomes (PROs) are used to assess HRQL in patients with IPF. Some were designed to assess the impact of pulmonary disease^7^, ^8^, ^9^ or specific respiratory symptoms,^10^, ^11^, ^12^ whereas others make a more general assessment of HRQL.^13^^,^^14^ Identifying factors that may influence decline in HRQL in patients with IPF may help in the development of strategies to preserve patients’ HRQL as the disease progresses.

The Idiopathic Pulmonary Fibrosis Prospective Outcomes (IPF-PRO) Registry (NCT01915511) is a prospective registry of patients with IPF conducted at 46 centers across the United States.^15^ One of the aims of this registry is to improve knowledge of the course of IPF and its impact on patients. In this analysis, we assessed trajectories of HRQL decline in the patients in the IPF-PRO Registry using a battery of respiratory-specific and global PROs. We also investigated the hypothesis that patient characteristics at enrollment would influence HRQL at enrollment and changes in HRQL during follow-up.

## Study Design and Methods

### Patients

Patients were eligible to participate in the IPF-PRO Registry if they had IPF that was diagnosed or confirmed at the enrolling center in the previous 6 months. Patients were enrolled at 46 sites across the United States (listed in e-Appendix 1) between June 2014 and October 2018. At enrollment, data were taken from patients’ medical records. Patients were then followed prospectively, with clinic visits routinely performed as part of clinical care approximately every 6 months, until the patient withdrew from the registry, died, or had a lung transplant. For this analysis, data were extracted on June 1, 2023. The study was approved by the Duke University institutional review board (Pro00046131). At each site (listed in e-Appendix 1), the protocol was approved by the relevant institutional review boards and/or local independent ethics committees before enrollment. All patients provided consent before their participation in the registry.

### Patient-Reported Outcomes

HRQL was assessed using the St. George’s Respiratory Questionnaire (SGRQ) (which contains 3 domains assessing symptoms, activity, and impact),^7^ the Cough and Sputum Assessment Questionnaire (CASA-Q) cough impact and cough symptoms domains,^12^ the Mental Component Summary (MCS) and Physical Component Summary (PCS) of the 12-item Short Form Survey (SF-12),^13^ and the EuroQol (EQ-5D-3L) score and EuroQol visual analog scale (VAS).^14^ These instruments have been shown to have adequate reliability, construct validity, and responsiveness in patients with IPF.^16^, ^17^, ^18^, ^19^, ^20^

SGRQ scores (total and for each domain), CASA-Q domain scores, and SF-12 MCS and PCS scores range from 0 to 100. The EuroQol score ranges from 0 to 1, and the VAS ranges from 0 to 100. Higher SGRQ scores indicate worse symptoms/HRQL, whereas lower CASA-Q, SF-12, and EuroQol scores indicate worse cough/HRQL. Minimal important changes to the patient (MICPs) in these scores have been estimated to be an increase of  ≥ 7 points for the SGRQ total score, an increase of ≥ 5 points for the SGRQ activity score, an increase of ≥ 7 points for the SGRQ impact score, and an increase of ≥ 8 points for the SGRQ symptoms score (data from patients with IPF)^21^; a decrease of  ≥ 11 points for the CASA-Q cough domain scores (data from patients with COPD)^22^; a decrease of ≥ 6 points for the SF-12 MCS score and a decrease of ≥ 5 points for the SF-12 PCS score (data from patients with IPF)^17^; and a decrease in ≥ 0.06 points for the EuroQol score^23^ and a decrease of ≥ 8 points for the EuroQol VAS (data from patients with lung cancer).^24^

### Modeling Trajectories of PROs

The number and timing of PRO measurements during follow-up were assessed descriptively. The mean trajectories of PRO scores over 48 months were estimated using a 2-component mixed joint model.^25^^,^^26^ One component was a mixed-effect linear model for the longitudinal scores, and the other was a survival model for time to a terminal event (defined as registry withdrawal, lung transplant, or death) over 48 months. The joint model fits the longitudinal and survival models simultaneously. Age, sex, FVC % predicted, diffusing capacity of the lungs for carbon monoxide (Dlco) % predicted, supplemental oxygen use only with activity, supplemental oxygen use at rest, and use of antifibrotic therapy (nintedanib or pirfenidone), all assessed at enrollment, were included as covariates in the longitudinal model. A patient-specific random intercept and a patient-specific random slope for time were also included. Missing values for covariates were handled using multiple imputation. Using the full conditional specification method, missing data were filled in 5 times to generate 5 complete data sets.^27^^,^^28^ One complete data set was randomly selected for modeling analyses because the modeling was computationally intensive and < 10% of patients had any missing data, making a single imputed data set sufficient.^29^ Age, BMI, FVC % predicted, Dlco % predicted, supplemental oxygen use only with activity, and supplemental oxygen use when at rest, all assessed at enrollment, were included as covariates in the survival model. The primary component of interest in this analysis was the longitudinal model; the survival model was included to account for nonrandom censoring of the longitudinal process that might otherwise have resulted in bias to the estimates of the trajectory parameters.

Associations between the clinical factors included in the longitudinal model and the trajectory of each PRO score were assessed in univariable and multivariable models. Where there was evidence of a nonlinear relationship between the score and time, a nonlinear time model based on natural cubic splines was used; otherwise, a linear time model was used. Before fitting the univariable and multivariable models, all covariate-time interactions were evaluated. Specifically, a model that included all possible interactions between time and covariates was fit, and interaction terms were eliminated, one at time, using backward selection, with an alpha criterion of .05.

The analysis cohort included patients who had ≥ 1 value for all the PROs at enrollment or during follow-up. Analyses were conducted using R version 4.2.3 or higher (R Foundation).

## Results

### Patients

**e-Figure 1:**
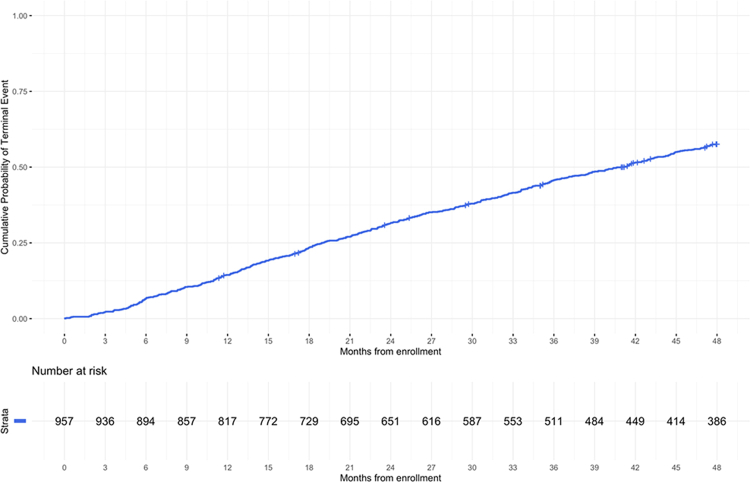
Kaplan Meier plot of time-to-terminal event used in the survival component of the joint model fits.

A total of 1,002 patients were enrolled in the registry. Of these, 957 patients were included in the analysis cohort. The characteristics of these patients at enrollment are shown in Table 1. Median age was 70.0 years (quartile [Q] 1, Q3: 65.0, 75.0), and 74.1% of patients were male. Median FVC was 73.1% predicted (Q1, Q3: 62.1, 84.0), and median Dlco was 42.5% predicted (Q1, Q3: 33.2, 51.9). Of the 957 patients in the analysis cohort, 546 experienced a terminal event within 48 months of enrollment. The Kaplan-Meier estimate for the terminal event rate at 48 months was 57.6% (95% CI, 54.3%-60.7%) (e-Fig 1).

**Table 1:** Characteristics of Analysis Cohort at Enrollment (N = 957)

Characteristic	Value
Age, y	70.0 (65.0, 75.0)
Male	709 (74.1)
BMI, kg/m^2^	29.0 (26.0, 32.3)
Smoking status	
Previous	632 (66.1)
Never	308 (32.2)
Active	16 (1.7)
Race/ethnicity	
White non-Hispanic	803 (91.9)
Black non-Hispanic	12 (1.4)
Other non-Hispanic	25 (2.9)
Hispanic	34 (3.9)
Diagnostic criteria for IPF^a^	
Definite	626 (65.4)
Probable	242 (25.3)
Possible	89 (9.3)
Diagnosis of IPF before referral to enrolling center	421 (44.1)
Family history of ILD (in parent, sibling, or grandparent)	179 (19.8)
Hospitalization in prior 12 mo	266 (28.9)
Respiratory hospitalization in prior 12 mo	164 (17.8)
Distance to enrolling center, miles	32.4 (13.8, 89.0)
Private insurance	579 (60.5)
FVC % predicted	73.1 (62.1, 84.0)
Dlco % predicted	42.5 (33.2, 51.9)
Oxygen use at rest	187 (19.5)
Oxygen use with activity only	148 (15.5)
Use of antifibrotic therapy	515 (53.8)

Data are presented as median (quartile 1, quartile 3) or No. (%). Not all patients provided data for all variables. Dlco = diffusing capacity of the lungs for carbon monoxide; ILD = interstitial lung disease; IPF = idiopathic pulmonary fibrosis.

a According to 2011 American Thoracic Society/European Respiratory Society/Japanese Respiratory Society/Asociación Latinoamericana de Tórax guidelines.^30^

### Patient-Reported Outcomes

**Figure 1:**
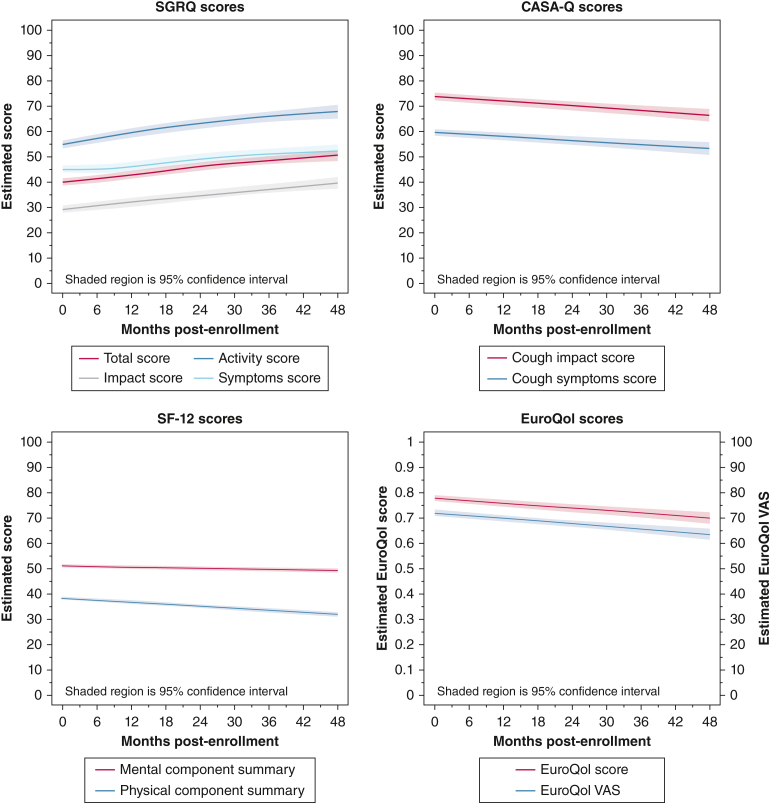
Estimated mean trajectories for each patient-reported outcome over 48 mo based on univariable models. Higher SGRQ scores indicate worse symptoms/health-related quality of life. Lower CASA-Q, SF-12, and EuroQol scores indicate worse cough/health-related quality of life. CASA-Q = Cough and Sputum Assessment Questionnaire; SF-12 = 12-item Short Form Survey; SGRQ = St. George’s Respiratory Questionnaire; VAS = visual analog scale.

At enrollment, 905, 925, 895, and 916 patients provided a value for the SGRQ, CASA-Q, SF-12, and EuroQol instruments, respectively. Median scores at enrollment are presented in Table 2. The median number of measurements per patient was 3.0 (Q1, Q3: 2.0, 5.0) over a median follow-up of 39.9 months (Q1, Q3: 18.8, 59.0). The median time between measurements was 6.4 months (Q1, Q3: 5.5, 8.3). All the PROs showed worsening over time (e-Tables 1-10, Fig 1). Changes in the SGRQ domain and total scores, SF-12 PCS, EuroQol score, and EuroQol VAS over 48 months exceeded published estimates for MICPs. For the SGRQ scores, there was evidence that the change over time was nonlinear (e-Table 1).

**Table 2:** Patient-Reported Outcome Scores at Enrollment

Patient-Reported Outcome	Value
SGRQ, No.	905
Total	39.4 (25.2, 53.3)
Activity	59.5 (38.4, 72.8)
Symptoms	43.2 (30.3, 60.5)
Impact	25.9 (13.8, 41.5)
CASA-Q, No.	925
Cough impact	78.1 (56.2, 93.8)
Cough symptoms	58.3 (41.7, 75.0)
SF-12, No.	895
MCS	53.7 (45.7, 59.2)
PCS	38.9 (31.1, 46.0)
EuroQol, No.	916
Score	0.8 (0.7, 1.0)
VAS	75 (60, 85)

Data are presented as median (quartile 1, quartile 3) unless otherwise specified. Higher SGRQ scores indicate worse symptoms/health-related quality of life. Lower CASA-Q, SF-12, and EuroQol scores indicate worse cough/health-related quality of life. CASA-Q = Cough and Sputum Assessment Questionnaire; MCS = Mental Component Summary; PCS = Physical Component Summary; SF-12 = 12-item Short Form Survey; SGRQ = St. George’s Respiratory Questionnaire; VAS = visual analog scale.

### Modeling of SGRQ Scores

**Figure 2:**
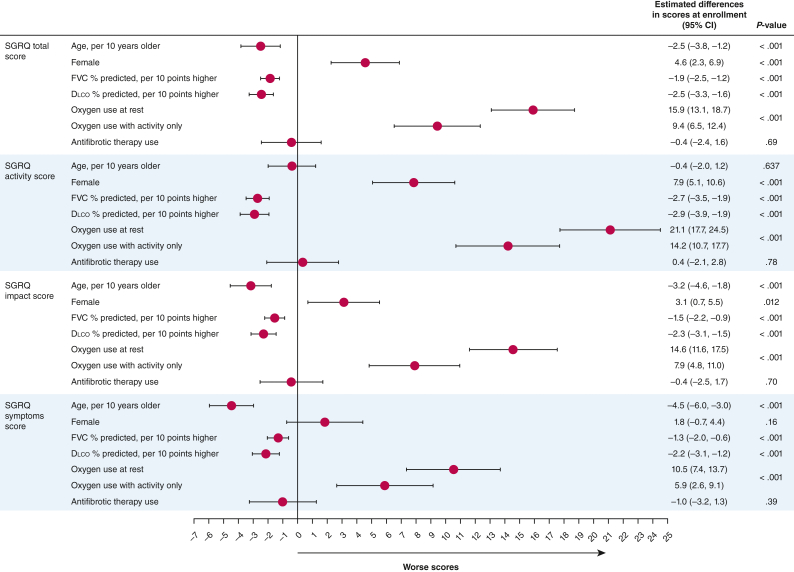
Estimated differences in SGRQ scores at enrollment based on multivariable models. Dlco = diffusing capacity of the lungs for carbon monoxide; SGRQ = St. George’s Respiratory Questionnaire.

**Figure 3:**
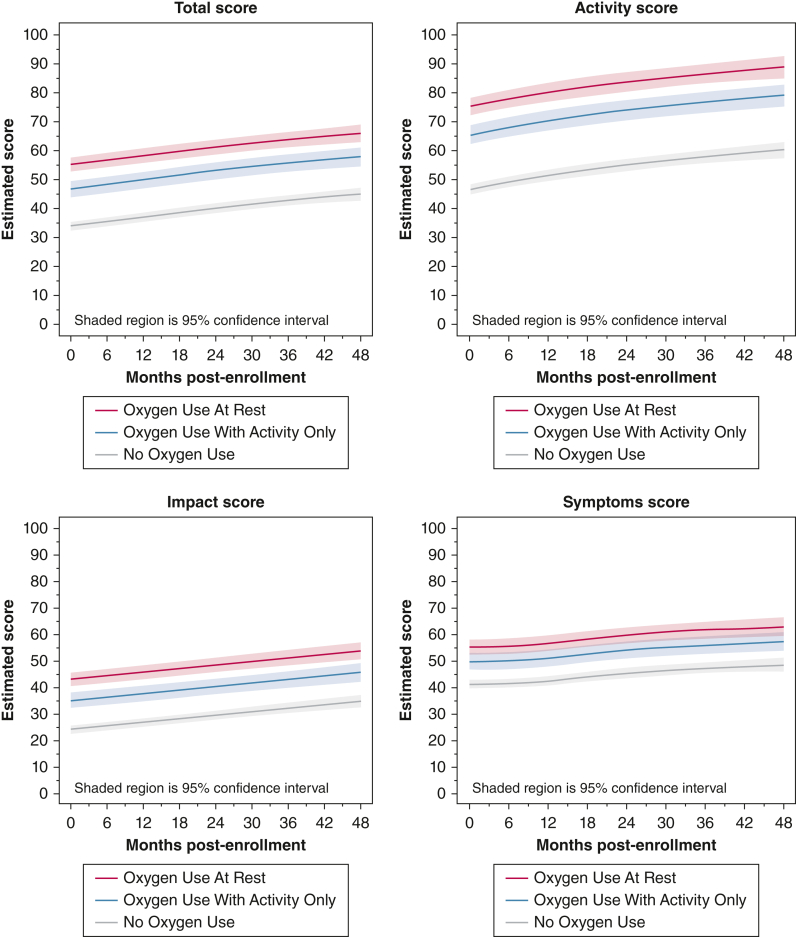
Estimated trajectories of St. George’s Respiratory Questionnaire scores by oxygen use at enrollment based on univariable models.

**Figure 4:**
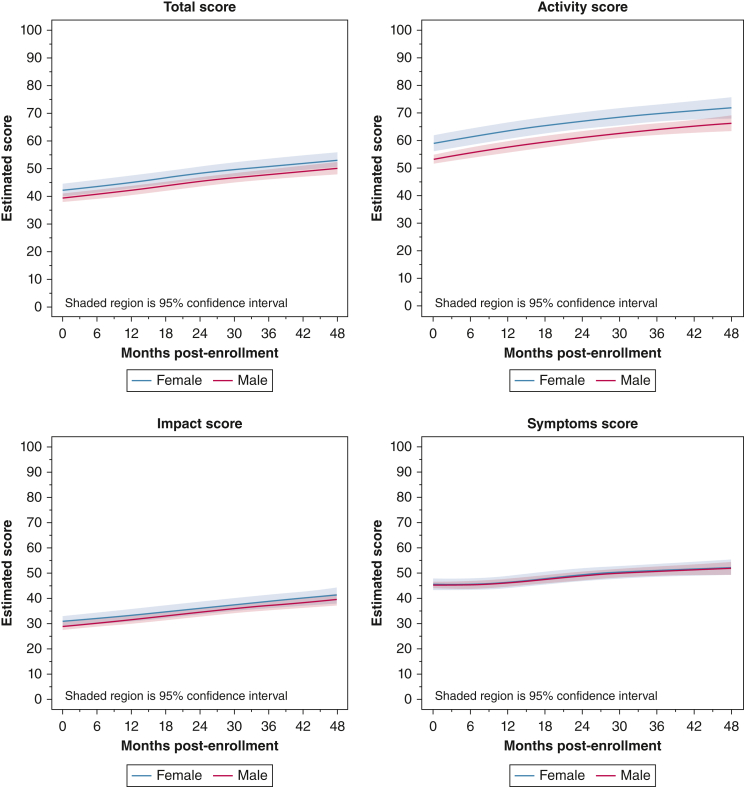
Estimated trajectories of St. George’s Respiratory Questionnaire scores by sex based on univariable models.

There were significant differences in SGRQ scores at enrollment across levels of clinical factors (Fig 2). Lower FVC % predicted, lower Dlco % predicted, use of supplemental oxygen, lower age, and female sex were independently associated with worse SGRQ total score at enrollment. The largest differences were observed with oxygen use: the estimated mean differences in SGRQ total score compared with patients not using oxygen were 15.9 for patients using oxygen at rest and 9.4 for patients using oxygen with activity only. There were no significant differences in SGRQ domain or total scores at enrollment by use of antifibrotic therapy. Estimated mean changes over 48 months were 10.8 (95% CI, 8.8-12.8), 7.0 (95% CI, 4.5-9.5), 13.1 (95% CI, 10.6-15.6) and 10.5 (95% CI, 8.3-12.6) for the SGRQ total, symptoms, activity, and impact scores, respectively. There were no significant differences in the trajectories of SGRQ domain or total scores over time across levels of clinical factors at enrollment, including oxygen use and sex (Figure 3, Figure 4).

### Modeling of CASA-Q Cough Symptoms and Impact Scores

**Figure 5:**
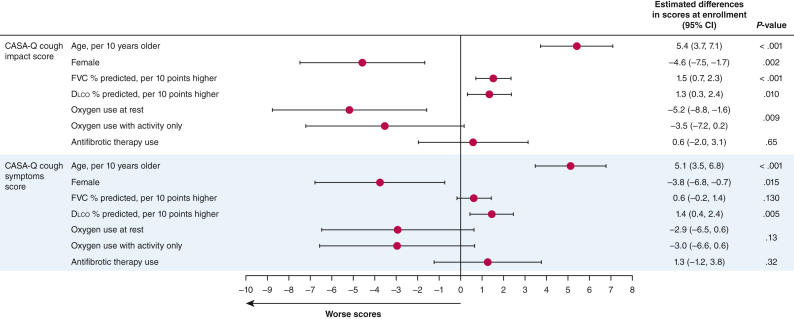
Estimated differences in CASA-Q cough impact and cough symptoms scores at enrollment based on multivariable models. CASA-Q = Cough and Sputum Assessment Questionnaire; Dlco = diffusing capacity of the lungs for carbon monoxide.

Younger age, female sex, and lower Dlco % predicted were independently associated with worse CASA-Q cough impact and symptoms scores at enrollment (Fig 5). Lower FVC % predicted and use of supplemental oxygen were independently associated with worse CASA-Q cough impact score at enrollment. Estimated mean changes over 48 months were −7.6 (95% CI, −10.0 to −5.1) and −6.5 (95% CI, −8.9 to −4.0) for the CASA-Q cough impact and symptoms scores, respectively. Compared with male patients, female patients had a smaller rate of decline in CASA-Q cough symptoms score over 12 months (difference, 1.4; 95% CI, 0.1-2.7; interaction *P* = .04). Other than for sex, there were no significant differences in the trajectories of CASA-Q cough impact scores over time across levels of clinical factors.

### Modeling of SF-12 Scores

**Figure 6:**
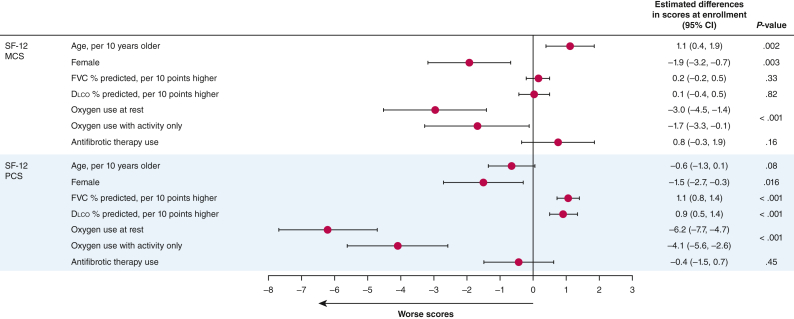
Estimated differences in SF-12 scores at enrollment based on multivariable models. Dlco = diffusing capacity of the lungs for carbon monoxide; MCS = Mental Component Summary; PCS = Physical Component Summary; SF-12 = 12-item Short Form Survey.

Younger age, female sex, and use of supplemental oxygen were independently associated with worse SF-12 MCS score at enrollment (Fig 6). Female sex, lower FVC % predicted, lower Dlco % predicted, and supplemental oxygen use were independently associated with worse SF-12 PCS score at enrollment. Estimated mean changes over 48 months were −2.1 (95% CI, −3.1 to −1.1) for the SF-12 MCS score and −7.0 (95% CI, −8.2 to −5.8) for the SF-12 PCS score. Patients with higher Dlco % predicted at enrollment had a smaller rate of decline in SF-12 MCS score over 12 months (difference, 0.2; 95% CI, 0.0-0.4; interaction *P* = .04) and in SF-12 PCS score over 12 months (difference, 0.2; 95% CI, 0.1-0.5; interaction *P* = .002). Other than this, there were no significant differences in the trajectories of SF-12 PCS scores over time across levels of clinical factors.

### Modeling of EuroQol Scores

**Figure 7:**
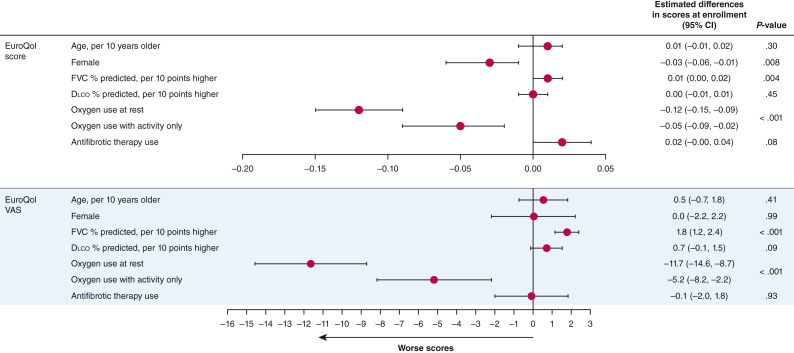
Estimated differences in EuroQol scores at enrollment based on multivariable models. Dlco = diffusing capacity of the lungs for carbon monoxide; VAS = visual analog scale.

Lower FVC % predicted and use of supplemental oxygen were independently associated with worse EuroQol and EuroQol VAS scores at enrollment (Fig 7). Female sex was independently associated with worse EuroQol score at enrollment. There were no significant differences in EuroQol score or EuroQol VAS score at enrollment by age, Dlco % predicted, or use of antifibrotic therapy. Estimated mean changes over 48 months were −0.08 (95% CI, −0.11 to −0.06) and −9.0 (95% CI, −11.3 to −6.7) for the EuroQol and EuroQol VAS scores, respectively. Patients with higher Dlco % predicted at enrollment had smaller rates of decline in EuroQol score and EuroQol VAS score over 12 months (difference, 0.01; 95% CI, 0.00-0.01; interaction *P* = .003; difference, 0.47; 95% CI, 0.06-0.88; interaction *P* = .024, respectively). Patients using oxygen at rest did not have a decline in EuroQol VAS score, whereas patients using oxygen with activity only or not using oxygen had a decline in EuroQol VAS score. Other than for Dlco and oxygen use, there were no significant differences in the trajectories of EuroQol scores or EuroQol VAS scores over time across levels of clinical factors.

## Discussion

Data from patients enrolled in the IPF-PRO Registry were used to evaluate trajectories of HRQL in patients with IPF. A range of PROs, assessing different aspects of HRQL, all showed worsening over 48 months. Changes in the SGRQ domain and total scores, SF-12 PCS, EuroQol score, and EuroQol VAS over 48 months exceeded published estimates for MICPs in these PROs. After adjustment for other covariates, lower age, female sex, lower FVC % predicted, lower Dlco % predicted, and use of supplemental oxygen were associated with worse respiratory-specific and global HRQL at enrollment, but not with variation in the trajectories of decline observed over time. These data support evidence from previous real-world studies showing that patients with IPF experience a marked decline in HRQL as the disease progresses^4^, ^5^, ^6^ and add to the literature on demographic and clinical factors influencing HRQL in patients with IPF.^2^^,^^31^, ^32^, ^33^, ^34^

Analyses of data from clinical trials and observational studies have shown associations between worse lung function and worse HRQL in patients with IPF.^16^^,^^31^^,^^33^^,^^35^^,^^36^ In our analysis, oxygen use at rest showed the strongest association with worse PRO scores at enrollment, with differences between patients using and not using oxygen exceeding published MICPs for the SGRQ, SF-12 PCS, and EuroQol scores. The difference in the SGRQ activity score was particularly striking, with a difference between patients using oxygen at rest vs not using oxygen of > 21 points, compared with an estimated MICP of 5 points.^21^ These data add to literature showing that patients with IPF whose disease has become so severe that they need to use supplemental oxygen have substantial impairment in HRQL^31^^,^^36^^,^^37^ and are in keeping with the findings of a qualitative study assessing the views of caregivers on the impact of IPF, which suggested that needing supplemental oxygen creates angst and prevents patients from living normal lives.^38^

In our study, female patients generally had worse HRQL at enrollment than male patients and retained worse HRQL throughout the follow-up period. This was observed for both respiratory-specific and global measures of HRQL. Consistent with this finding, baseline data from the INPULSIS trials^39^, ^40^, ^41^, ^42^ and from a French registry in patients with IPF^34^ showed that mean SGRQ total scores were worse in female participants than male participants (44.3 vs 38.3 and 48.5 vs 41.5, respectively). In addition, pooled data from 2 tertiary care centers in the United States (N = 1,263) showed that a mean score on the University of California San Diego Shortness of Breath Questionnaire was worse in female participants than male participants (49 vs 35 points).^32^ However, among patients in the Swedish IPF registry (N = 292), no significant difference was found between male and female patients in mean score on the King’s Brief Interstitial Lung Disease questionnaire (53.6 vs 54.3, respectively).^43^ The reasons behind the observation of worse HRQL in female patients than male patients are not understood, but it has been suggested that there may be differences in the disease course or care of patients with ILDs between the sexes.^44^

We found that younger patients generally had worse HRQL at enrollment and over the course of the registry than older patients, after adjusting for covariates including lung function. A systematic review of 134 studies also found that younger patients had worse HRQL than older patients, based on the SGRQ, 36-item Short Form Survey, and King’s Brief Interstitial Lung Disease questionnaire.^2^ The reasons why younger patients with IPF report worse HRQL than older patients are not well understood, but may be related to a worse perception of disease severity among younger patients who experience symptoms or functional limitations. Although interventions (eg, symptom relief, pulmonary rehabilitation, psychosocial support) should be considered as part of care for all patients with IPF,^45^, ^46^, ^47^ these data suggest that interventions aimed at preserving HRQL may be of particular benefit for certain groups of patients. Further evidence is needed to inform the strategies that are most effective in preserving HRQL in particular groups of patients with IPF.

Strengths of our study include a large sample size and the use of a joint model that accounted for nonrandom censoring of the longitudinal process that might otherwise have biased estimates of the association between the clinical factors and PROs. Although statistically significant, differences in PROs at enrollment and in the rates of change in PROs across some levels of clinical characteristics were small. Limitations of our analyses include missing data, which may be informative in that patients with worse disease may have attended fewer clinic appointments and so completed fewer PROs. The PROs assessed in our study were not developed specifically for use with patients with IPF. PROs developed in patients with ILD, such as the Living with Pulmonary Fibrosis questionnaire, which has been shown to be responsive to changes in health status in patients with ILDs,^48^^,^^49^ were not included in the IPF-PRO Registry but may provide a greater understanding of the impact of IPF on patients’ HRQL in future studies.

## Interpretation

These data from the IPF-PRO Registry suggest that for several PROs, the deterioration in scores over 48 months exceeded estimated MICPs. Younger age, female sex, worse lung function, and use of supplemental oxygen were associated with worse HRQL at enrollment, but not with different trajectories of PROs during follow-up. These findings highlight the importance of interventions in clinical practice to preserve HRQL in patients with IPF and identify populations of particular interest for future studies.

A graphical abstract summarizing these data is available at https://www.usscicomms.com/respiratory/IPF-PRORegistry_TrajectoriesOfHRQL.

## Funding/Support

The IPF-PRO/ILD-PRO Registry is supported by 10.13039/100001003Boehringer Ingelheim Pharmaceuticals, Inc.

## Financial/Nonfinancial Disclosures

The authors have reported to *CHEST Pulmonary* the following: M. L. N., J. L. T., and L. D. S. are faculty members of the Duke Clinical Research Institute (DCRI), which receives funding support from Boehringer Ingelheim Pharmaceuticals, Inc to coordinate the IPF-PRO/ILD-PRO Registry. J. L. T. also reports grants from AstraZeneca and CareDx and has participated on advisory boards for Altavant Sciences, Natera, Sanofi, and Theravance. P. L. and A. L. O. are employees of Boehringer Ingelheim Pharmaceuticals, Inc.

## Supplementary Data

e-Online Data

e-Online Data

e-Online Data

e-Online Data
