# Supplementary material for: Trajectories of Health-Related Quality of Life in Patients With Idiopathic Pulmonary Fibrosis
Source: CHEST Pulm. 2024 Dec 27;3(4):100133. doi: 10.1016/j.chpulm.2024.100133 (PMC13417800; doi:10.1016/j.chpulm.2024.100133)
Supplement: e-Online Data [file mmc1.docx]

**Supplementary Material**

**Trajectories of health-related quality of life in patients with idiopathic pulmonary fibrosis**

Megan L Neely, PhD,^1,2^ Jamie L Todd, MD,^1,2^ Laurie D Snyder, MD,^1,2^ Peide Li, PhD,^3^ Amy L Olson, MD^3^ on behalf of the IPF-PRO Registry investigators

^1^Duke Clinical Research Institute, Durham, NC, USA; ^2^Duke University Medical Center, Durham, NC, USA; ^3^Boehringer Ingelheim Pharmaceuticals, Inc., Ridgefield, CT, USA.

**e-Appendix 1**

**Principal investigators and enrolling centers in the IPF-PRO Registry**

Albert Baker, Lynchburg Pulmonary Associates, Lynchburg, VA; Scott Beegle, Albany Medical Center, Albany, NY; John A Belperio, University of California Los Angeles, Los Angeles, CA; Rany Condos, NYU Medical Center, New York, NY; Francis Cordova, Temple University, Philadelphia, PA; Brian Southern (formerly Daniel A Culver), Cleveland Clinic, Cleveland, OH; Daniel Dilling, Loyola University Health System, Maywood, IL; John Fitzgerald (formerly Leann Silhan), UT Southwestern Medical Center, Dallas, TX; Kevin R Flaherty, University of Michigan, Ann Arbor, MI; Kevin Gibson, University of Pittsburgh, Pittsburgh, PA; Mridu Gulati, Yale School of Medicine, New Haven, CT; Kalpalatha Guntupalli, Baylor College of Medicine, Houston, TX; Nishant Gupta, University of Cincinnati Medical Center, Cincinnati, OH; Amy Hajari Case, Piedmont Healthcare, Atlanta, GA; David Hotchkin, The Oregon Clinic, Portland, OR; Tristan J Huie, National Jewish Health, Denver, CO; Robert J Kaner, Weill Cornell Medical College, New York, NY; Hyun J Kim, University of Minnesota, Minneapolis, MN; Lisa H Lancaster (formerly Mark Steele), Vanderbilt University Medical Center, Nashville, TN; Joseph A Lasky, Tulane University, New Orleans, LA; Doug Lee, Wilmington Health and PMG Research, Wilmington, NC; Timothy Liesching, Lahey Clinic, Burlington, MA; Randolph Lipchik, Froedtert & The Medical College of Wisconsin Community Physicians, Milwaukee, WI; Jason Lobo, UNC Chapel Hill, Chapel Hill, NC; Tracy R Luckhardt (formerly Joao A de Andrade), University of Alabama at Birmingham, Birmingham, AL; Yolanda Mageto (formerly Howard Huang), Baylor University Medical Center at Dallas, Dallas, TX; Marta Kokoszynska (formerly Yolanda Mageto, Prema Menon), Vermont Lung Center, Colchester, VT; Lake Morrison, Duke University Medical Center, Durham, NC; Andrew Namen, Wake Forest University, Winston Salem, NC; Namita Sood (formerly Justin M Oldham), University of California, Davis, Sacramento, CA; Tessy Paul, University of Virginia, Charlottesville, VA; David Zhang (formerly Anna Podolanczuk, David Lederer, Nina M Patel), Columbia University Medical Center/New York Presbyterian Hospital, New York, NY; Mary Porteous (formerly Maryl Kreider), University of Pennsylvania, Philadelphia, PA; Rishi Raj (formerly Paul Mohabir), Stanford University, Stanford, CA; Murali Ramaswamy, PulmonIx LLC, Greensboro, NC; Tonya Russell, Washington University, St. Louis, MO; Paul Sachs, Pulmonary Associates of Stamford, Stamford, CT; Zeenat Safdar, Houston Methodist Lung Center, Houston, TX; Shirin Shafazand (formerly Marilyn Glassberg), University of Miami, Miami, FL; Ather Siddiqi (formerly Wael Asi), Renovatio Clinical, The Woodlands, TX; Reginald Fowler (formerly Barry Sigal), Salem Chest and Southeastern Clinical Research Center, Winston Salem, NC; Mary E Strek (formerly Imre Noth), University of Chicago, Chicago, IL; Rania Abdallah (formerly Jesse Roman, Sally Suliman, Hiram Rivas-Perez), University of Louisville, Louisville, KY; Jeremy Tabak, South Miami Hospital, South Miami, FL; Rajat Walia, St. Joseph’s Hospital, Phoenix, AZ; Timothy PM Whelan, Medical University of South Carolina, Charleston, SC.
