# Supplementary material for: Trajectories of Health-Related Quality of Life in Patients With Idiopathic Pulmonary Fibrosis
Source: CHEST Pulm. 2024 Dec 27;3(4):100133. doi: 10.1016/j.chpulm.2024.100133 (PMC13417800; doi:10.1016/j.chpulm.2024.100133)
Supplement: e-Online Data [file mmc3.docx]

**Supplementary Material**

**Trajectories of health-related quality of life in patients with idiopathic pulmonary fibrosis**

Megan L Neely, PhD,^1,2^ Jamie L Todd, MD,^1,2^ Laurie D Snyder, MD,^1,2^ Peide Li, PhD,^3^ Amy L Olson, MD^3^ on behalf of the IPF-PRO Registry investigators

^1^Duke Clinical Research Institute, Durham, NC, USA; ^2^Duke University Medical Center, Durham, NC, USA; ^3^Boehringer Ingelheim Pharmaceuticals, Inc., Ridgefield, CT, USA.

**e-Figure 1.** Kaplan Meier plot of time-to-terminal event used in the survival component of the joint model fits.
