# Supplementary material for: Trajectories of Health-Related Quality of Life in Patients With Idiopathic Pulmonary Fibrosis
Source: CHEST Pulm. 2024 Dec 27;3(4):100133. doi: 10.1016/j.chpulm.2024.100133 (PMC13417800; doi:10.1016/j.chpulm.2024.100133)
Supplement: e-Online Data [file mmc4.docx]

**Supplementary Material**

**Trajectories of health-related quality of life in patients with idiopathic pulmonary fibrosis**

Megan L Neely, PhD,^1,2^ Jamie L Todd, MD,^1,2^ Laurie D Snyder, MD,^1,2^ Peide Li, PhD,^3^ Amy L Olson, MD^3^ on behalf of the IPF-PRO Registry investigators

^1^Duke Clinical Research Institute, Durham, NC, USA; ^2^Duke University Medical Center, Durham, NC, USA; ^3^Boehringer Ingelheim Pharmaceuticals, Inc., Ridgefield, CT, USA.

e-Table 1. Joint model fits for SGRQ total score trajectory.

|  | **Univariable model*** | | | | **Multivariable model*** | | | |
| --- | --- | --- | --- | --- | --- | --- | --- | --- |
| **Trajectory** | **Trajectory intercept**  **(95% CI)** | **P- value** | **Trajectory net change (95% CI)^†^** | **P-value** | **Trajectory intercept (95% CI)** | **P-value** | **Trajectory net Change (95% CI)^†^** | **P-Value** |
| Time | --- | --- |  | <0.001 | --- | --- |  | <0.001 |
| Over 12 months | --- | --- | 2.9 (1.8, 3.9) |  | --- | --- | 3.1 (2.0, 4.1) |  |
| Over 24 months | --- | --- | 6.1 (4.9, 7.2) |  | --- | --- | 6.3 (5.1, 7.4) |  |
| Over 36 months | --- | --- | 8.6 (7.4, 9.8) |  | --- | --- | 8.9 (7.7, 10.1) |  |
| Over 48 months | --- | --- | 10.8 (8.8, 12.8) |  | --- | --- | 11.1 (9.2, 13.1) |  |
| **Covariates** | **Trajectory intercept**  **difference (95% CI)** | **P- value** | **Trajectory net change**  **difference (95% CI)** | **P-value^‡^** | **Trajectory intercept**  **difference (95% CI)** | **P-value** | **Trajectory net change**  **difference (95% CI)** | **P-value^‡^** |
| Age (per 10 years higher) | −2.7 (−4.2, −1.1) | <0.001 | --- | --- | −2.5 (−3.8, −1.2) | <0.001 | --- | --- |
| Female vs male sex | 2.9 (0.2, 5.7) | 0.037 | --- | --- | 4.6 (2.3, 6.9) | <0.001 | --- | --- |
| FVC % predicted (per 10 units higher) | −3.8 (−4.5, −3.2) | <0.001 | --- | --- | −1.9 (−2.5, −1.2) | <0.001 | --- | --- |
| DLco % predicted (per 10 units higher) | −5.0 (−5.7, −4.2) | <0.001 | --- | --- | −2.5 (−3.3, −1.6) | <0.001 | --- | --- |
| Supplemental oxygen use |  | <0.001 | --- | --- |  | <0.001 | --- | --- |
| At rest vs none^§^ | 21.2 (18.4, 24.0) |  | --- | --- | 15.9 (13.1, 18.7) |  | --- | --- |
| With activity only vs none | 12.9 (9.9, 16.0) |  | --- | --- | 9.4 (6.5, 12.4) |  | --- | --- |
| Antifibrotic drug use | 1.4 (−1.0, 3.8) | 0.249 | --- | --- | −0.4 (−2.4, 1.6) | 0.688 | --- | --- |

*Univariable models included only time or only time and covariate in longitudinal component of joint model; multivariable model included time and all covariates in longitudinal component.
^†^Net change reported instead of trajectory slope estimate because there was evidence of a non-linear relationship with time (p=0.036).
^‡^Covariate-time interaction p-value (if applicable).
^§^Included all subjects using oxygen at rest, regardless of whether they used oxygen with activity.

e-Table 2. Joint model fits for SGRQ activity score trajectory.

|  | **Univariable model*** | | | | **Multivariable model*** | | | |
| --- | --- | --- | --- | --- | --- | --- | --- | --- |
| **Trajectory** | **Trajectory intercept**  **(95% CI)** | **P- value** | **Trajectory net change (95% CI)^†^** | **P-value** | **Trajectory intercept (95% CI)** | **P-value** | **Trajectory net Change (95% CI)^†^** | **P-Value** |
| Time | --- | --- |  | <0.001 | --- | --- |  | <0.001 |
| Over 12 months | --- | --- | 4.6 (3.3, 6.0) |  | --- | --- | 4.9 (3.6, 6.3) |  |
| Over 24 months | --- | --- | 8.2 (6.8, 9.7) |  | --- | --- | 8.6 (7.1, 10.1) |  |
| Over 36 months | --- | --- | 10.9 (9.4, 12.5) |  | --- | --- | 11.4 (9.9, 12.9) |  |
| Over 48 months | --- | --- | 13.1 (10.6, 15.6) |  | --- | --- | 13.8 (11.3, 16.2) |  |
| **Covariates** | **Trajectory intercept**  **difference (95% CI)** | **P- value** | **Trajectory net change**  **difference (95% CI)** | **P-value^‡^** | **Trajectory intercept**  **difference (95% CI)** | **P-value** | **Trajectory net change**  **difference (95% CI)** | **P-value^‡^** |
| Age (per 10 years higher) | −0.7 (−2.6, 1.2) | 0.469 | --- | --- | −0.4 (−2.0, 1.2) | 0.637 | --- | --- |
| Female vs male sex | 5.8 (2.4, 9.2) | <0.001 | --- | --- | 7.9 (5.1, 10.6) | <0.001 | --- | --- |
| FVC % predicted (per 10 units higher) | −5.0 (−5.8, −4.2) | <0.001 | --- | --- | −2.7 (−3.5, −1.9) | <0.001 | --- | --- |
| DLco % predicted (per 10 units higher) | −6.4 (−7.4, −5.5) | <0.001 | --- | --- | −2.9 (−3.9, −1.9) | <0.001 | --- | --- |
| Supplemental oxygen use |  | <0.001 | --- | --- |  | <0.001 | --- | --- |
| At rest vs none^§^ | 28.4 (25.0, 31.7) |  | --- | --- | 21.1 (17.7, 24.5) |  | --- | --- |
| With activity only vs none | 18.7 (15.1, 22.4) |  | --- | --- | 14.2 (10.7, 17.7) |  | --- | --- |
| Antifibrotic drug use | 2.4 (−0.6, 5.4) | 0.116 | --- | --- | 0.4 (−2.1, 2.8) | 0.778 | --- | --- |

*Univariable models included only time or only time and covariate in longitudinal component of joint model; multivariable model included time and all covariates in longitudinal component.
^†^Net change reported instead of trajectory slope estimate because there was evidence of a non-linear relationship with time (p=0.048).
^‡^Covariate-time interaction p-value (if applicable).
^§^Included all subjects using oxygen at rest, regardless of whether they used oxygen with activity.

e-Table 3. Joint model fits for SGRQ impact score trajectory.

|  | **Univariable model*** | | | | **Multivariable model*** | | | |
| --- | --- | --- | --- | --- | --- | --- | --- | --- |
| **Trajectory** | **Trajectory intercept**  **(95% CI)** | **P- value** | **Trajectory net change (95% CI)^†^** | **P-value** | **Trajectory intercept (95% CI)** | **P-value** | **Trajectory net Change (95% CI)^†^** | **P-Value** |
| Time | --- | --- |  | <0.001 | --- | --- |  | <0.001 |
| Over 12 months | --- | --- | 2.5 (1.3, 3.7) |  | --- | --- | 2.7 (1.6, 3.9) |  |
| Over 24 months | --- | --- | 5.4 (4.1, 6.7) |  | --- | --- | 5.6 (4.3, 6.9) |  |
| Over 36 months | --- | --- | 8.0 (6.7, 9.3) |  | --- | --- | 8.3 (7.0, 9.6) |  |
| Over 48 months | --- | --- | 10.5 (8.3, 12.6) |  | --- | --- | 10.9 (8.8, 13.0) |  |
| **Covariates** | **Trajectory intercept**  **difference (95% CI)** | **P- value** | **Trajectory net change**  **difference (95% CI)** | **P-value^‡^** | **Trajectory intercept**  **difference (95% CI)** | **P-value** | **Trajectory net change**  **difference (95% CI)** | **P-value^‡^** |
| Age (per 10 years higher) | −3.3 (−4.8, −1.7) | <0.001 | --- | --- | −3.2 (−4.6, −1.8) | <0.001 | --- | --- |
| Female vs male sex | 1.6 (−1.2, 4.4) | 0.254 | --- | --- | 3.1 (0.7, 5.5) | 0.012 | --- | --- |
| FVC % predicted (per 10 units higher) | −3.4 (−4.1, −2.8) | <0.001 | --- | --- | −1.5 (−2.2, −0.9) | <0.001 | --- | --- |
| DLco % predicted (per 10 units higher) | −4.5 (−5.3, −3.7) | <0.001 | --- | --- | −2.3 (−3.1, −1.5) | <0.001 | --- | --- |
| Supplemental oxygen use |  | <0.001 | --- | --- |  | <0.001 | --- | --- |
| At rest vs none^§^ | 19.2 (16.3, 22.0) |  | --- | --- | 14.6 (11.6, 17.5) |  | --- | --- |
| With activity only vs none | 11.0 (7.9, 14.2) |  | --- | --- | 7.9 (4.8, 11.0) |  | --- | --- |
| Antifibrotic drug use | 1.4 (−1.1, 3.8) | 0.272 | --- | --- | −0.4 (−2.5, 1.7) | 0.700 | --- | --- |

*Univariable models included only time or only time and covariate in longitudinal component of joint model; multivariable model included time and all covariates in longitudinal component.
^†^Net change reported instead of trajectory slope estimate because there was evidence of a non-linear relationship with time (p=0.027).
^‡^Covariate-time interaction p-value (if applicable).
^§^Included all subjects using oxygen at rest, regardless of whether they used oxygen with activity.

e-Table 4. Joint model fits for SGRQ symptoms score trajectory.

|  | **Univariable model*** | | | | **Multivariable model*** | | | |
| --- | --- | --- | --- | --- | --- | --- | --- | --- |
| **Trajectory** | **Trajectory intercept**  **(95% CI)** | **P- value** | **Trajectory net change (95% CI)^†^** | **P-value** | **Trajectory intercept (95% CI)** | **P-value** | **Trajectory net Change (95% CI)^†^** | **P-Value** |
| Time | --- | --- |  | <0.001 | --- | --- |  | <0.001 |
| Over 12 months | --- | --- | 1.0 (−0.4, 2.3) |  | --- | --- | 1.3 (−0.1, 2.7) |  |
| Over 24 months | --- | --- | 3.9 (2.4, 5.4) |  | --- | --- | 4.3 (2.8, 5.8) |  |
| Over 36 months | --- | --- | 5.8 (4.3, 7.4) |  | --- | --- | 6.3 (4.7, 7.8) |  |
| Over 48 months | --- | --- | 7.0 (4.5, 9.5) |  | --- | --- | 7.6 (5.1, 10.1) |  |
| **Covariates** | **Trajectory intercept**  **difference (95% CI)** | **P- value** | **Trajectory net change**  **difference (95% CI)** | **P-value^‡^** | **Trajectory intercept**  **difference (95% CI)** | **P-value** | **Trajectory net change**  **difference (95% CI)** | **P-value^‡^** |
| Age (per 10 years higher) | −4.5 (−6.1, −3.0) | <0.001 | --- | --- | −4.5 (−6.0, −3.0) | <0.001 | --- | --- |
| Female vs male sex | 0.4 (−2.4, 3.2) | 0.792 | --- | --- | 1.8 (−0.7, 4.4) | 0.161 | --- | --- |
| FVC % predicted (per 10 units higher) | −3.0 (−3.7, −2.4) | <0.001 | --- | --- | −1.3 (−2.0, −0.6) | <0.001 | --- | --- |
| DLco % predicted (per 10 units higher) | −3.8 (−4.6, −3.0) | <0.001 | --- | --- | −2.2 (−3.1, −1.2) | <0.001 | --- | --- |
| Supplemental oxygen use |  | <0.001 | --- | --- |  | <0.001 | --- | --- |
| At rest vs none^§^ | 14.4 (11.3, 17.4) |  | --- | --- | 10.5 (7.4, 13.7) |  | --- | --- |
| With activity only vs none | 8.7 (5.4, 12.0) |  | --- | --- | 5.9 (2.6, 9.1) |  | --- | --- |
| Antifibrotic drug use | 0.7 (−1.8, 3.1) | 0.603 | --- | --- | −1.0 (−3.2, 1.3) | 0.390 | --- | --- |

*Univariable models included only time or only time and covariate in longitudinal component of joint model; multivariable model included time and all covariates in longitudinal component.
^†^Net change reported instead of trajectory slope estimate because there was evidence of a non-linear relationship with time (p<0.001).
^‡^Covariate-time interaction p-value (if applicable).
^§^Included all subjects using oxygen at rest, regardless of whether they used oxygen with activity.

e-Table 5. Joint model fits for CASA-Q cough impact score trajectory.

|  | **Univariable model*** | | | | **Multivariable model*** | | | |
| --- | --- | --- | --- | --- | --- | --- | --- | --- |
| **Trajectory** | **Trajectory intercept**  **(95% CI)** | **P- value** | **Trajectory slope (95% CI)^†^** | **P-value** | **Trajectory intercept (95% CI)** | **P-value** | **Trajectory slope (95% CI)^†^** | **P-Value** |
| Time | 73.9 (72.5, 75.3) | <0.001 | −1.9 (−2.5, −1.3) | <0.001 | 21.7 (9.0, 34.3) | <0.001 | −2.0 (−2.6, −1.4) | <0.001 |
| **Covariates** | **Trajectory intercept**  **difference (95% CI)** | **P- value** | **Trajectory slope**  **difference (95% CI)** | **P-value^‡^** | **Trajectory intercept**  **difference (95% CI)** | **P-value** | **Trajectory slope**  **difference (95% CI)** | **P-value^‡^** |
| Age (per 10 years higher) | 5.7 (4.0, 7.4) | <0.001 | --- | --- | 5.4 (3.7, 7.1) | <0.001 | --- | --- |
| Female vs male sex | −3.2 (−6.2, −0.2) | 0.038 | --- | --- | −4.6 (−7.5, −1.7) | 0.002 | --- | --- |
| FVC % predicted (per 10 units higher) | 2.6 (1.9, 3.4) | <0.001 | --- | --- | 1.5 (0.7, 2.3) | <0.001 | --- | --- |
| DLco % predicted (per 10 units higher) | 2.5 (1.6, 3.4) | <0.001 | --- | --- | 1.3 (0.3, 2.4) | 0.010 | --- | --- |
| Supplemental oxygen use |  | <0.001 | --- | --- |  | 0.009 | --- | --- |
| At rest vs none^§^ | −8.1 (−11.6, −4.6) |  | --- | --- | −5.2 (−8.8, −1.6) |  | --- | --- |
| With activity only vs none | −5.9 (−9.6, −2.1) |  | --- | --- | −3.5 (−7.2, 0.2) |  | --- | --- |
| Antifibrotic drug use | −0.6 (−3.3, 2.1) | 0.654 | --- | --- | 0.6 (−2.0, 3.1) | 0.653 | --- | --- |

*Univariable models included only time or only time and covariate in longitudinal component of joint model; multivariable model included time and all covariates in longitudinal component.
^†^Trajectory slope estimate reported because there was no evidence of a non-linear relationship with time (p>0.99); trajectory slope estimates reported per 12 months change in HRQL.
^‡^Covariate-time interaction p-value (if applicable).
^§^Included all subjects using oxygen at rest, regardless of whether they used oxygen with activity.

Net change over 48 months (95% CI) was −7.6 (−10.0, −5.1) in the univariable model and −7.9 (−10.3, −5.5) in the multivariable model.

e-Table 6. Joint model fits for CASA-Q cough symptoms score trajectory.

|  | **Univariable model*** | | | | **Multivariable model*** | | | |
| --- | --- | --- | --- | --- | --- | --- | --- | --- |
| **Trajectory** | **Trajectory intercept**  **(95% CI)** | **P- value** | **Trajectory slope (95% CI)^†^** | **P-value** | **Trajectory intercept (95% CI)** | **P-value** | **Trajectory slope (95% CI)^†^** | **P-Value** |
| Time | 59.6 (58.2, 60.9) | <0.001 | −1.6 (−2.2, −1.0) | <0.001 | 14.4 (2.0, 26.8) | 0.023 | −2.1 (−2.8, −1.4) | <0.001 |
| **Covariates** | **Trajectory intercept**  **difference (95% CI)** | **P- value** | **Trajectory slope**  **difference (95% CI)** | **P-value^‡^** | **Trajectory intercept**  **difference (95% CI)** | **P-value** | **Trajectory slope**  **difference (95% CI)** | **P-value^‡^** |
| Age (per 10 years higher) | 5.1 (3.5, 6.8) | <0.001 | --- | --- | 5.1 (3.5, 6.8) | <0.001 | --- | --- |
| Female vs male sex | −2.7 (−5.8, 0.4) | 0.087 | 1.3 (0.0, 2.6) | 0.050 | −3.8 (−6.8, −0.7) | 0.015 | 1.4 (0.1, 2.7) | 0.040 |
| FVC % predicted (per 10 units higher) | 1.7 (0.9, 2.4) | <0.001 | --- | --- | 0.6 (−0.2, 1.4) | 0.130 | --- | --- |
| DLco % predicted (per 10 units higher) | 1.9 (1.1, 2.8) | <0.001 | --- | --- | 1.4 (0.4, 2.4) | 0.005 | --- | --- |
| Supplemental oxygen use |  | 0.003 | --- | --- |  | 0.125 | --- | --- |
| At rest vs none^§^ | −4.8 (−8.2, −1.4) |  | --- | --- | −2.9 (−6.5, 0.6) |  | --- | --- |
| With activity only vs none | −4.6 (−8.2, −1.0) |  | --- | --- | −3.0 (−6.6, 0.6) |  | --- | --- |
| Antifibrotic drug use | 0.3 (−2.3, 2.8) | 0.836 | --- | --- | 1.3 (−1.2, 3.8) | 0.321 | --- | --- |

*Univariable models included only time or only time and covariate in longitudinal component of joint model; multivariable model included time and all covariates in longitudinal component.
^†^Trajectory slope estimate reported because there was no evidence of a non-linear relationship with time (p=0.52); trajectory slope estimates reported per 12 months change in HRQL.
^‡^Covariate-time interaction p-value (if applicable).
^§^Included all subjects using oxygen at rest, regardless of whether they used oxygen with activity.

Net change over 48 months (95% CI) was −6.5 (−8.9, −4.0) in the univariable model and −8.3 (−11.1, −5.4) in the multivariable model.

e-Table 7. Joint model fits for SF-12 MCS score trajectory.

|  | **Univariable model*** | | | | **Multivariable model*** | | | |
| --- | --- | --- | --- | --- | --- | --- | --- | --- |
| **Trajectory** | **Trajectory intercept**  **(95% CI)** | **P- value** | **Trajectory slope (95% CI)^†^** | **P-value** | **Trajectory intercept (95% CI)** | **P-value** | **Trajectory slope (95% CI)^†^** | **P-Value** |
| Time | 52.1 (51.5, 52.7) | <0.001 | −0.5 (−0.8, −0.3) | <0.001 | 43.5 (37.9, 49.0) | <0.001 | −1.5 (−2.5, −0.6) | <0.001 |
| **Covariates** | **Trajectory intercept**  **difference (95% CI)** | **P- value** | **Trajectory slope**  **difference (95% CI)** | **P-value^‡^** | **Trajectory intercept**  **difference (95% CI)** | **P-value** | **Trajectory slope**  **difference (95% CI)** | **P-value^‡^** |
| Age (per 10 years higher) | 1.0 (0.3, 1.8) | 0.005 | --- | --- | 1.1 (0.4, 1.9) | 0.002 | --- | --- |
| Female vs male sex | −1.8 (−3.0, −0.5) | 0.006 | --- | --- | −1.9 (−3.2, −0.7) | 0.003 | --- | --- |
| FVC % predicted (per 10 units higher) | 0.5 (0.2, 0.8) | 0.004 | --- | --- | 0.2 (−0.2, 0.5) | 0.325 | --- | --- |
| DLco % predicted (per 10 units higher) | 0.4 (−0.0, 0.8) | 0.070 | 0.2 (−0.0, 0.4) | 0.063 | 0.1 (−0.4, 0.5) | 0.821 | 0.2 (0.0, 0.4) | 0.035 |
| Supplemental oxygen use |  | <0.001 | --- | --- |  | <0.001 | --- | --- |
| At rest vs none^§^ | −3.3 (−4.7, −1.8) |  | --- | --- | −3.0 (−4.5, −1.4) |  | --- | --- |
| With activity only vs none | −1.9 (−3.5, −0.4) |  | --- | --- | −1.7 (−3.3, −0.1) |  | --- | --- |
| Antifibrotic drug use | 0.5 (−0.6, 1.6) | 0.370 | --- | --- | 0.8 (−0.3, 1.9) | 0.162 | --- | --- |

*Univariable models included only time or only time and covariate in longitudinal component of joint model; multivariable model included time and all covariates in longitudinal component.
^†^Trajectory slope estimate reported because there was no evidence of a non-linear relationship with time (p=0.29); trajectory slope estimates reported per 12 months change in HRQL.
^‡^Covariate-time interaction p-value (if applicable).
^§^Included all subjects using oxygen at rest, regardless of whether they used oxygen with activity.

Net change over 48 months (95% CI) was −2.1 (−3.1, −1.1) in the univariable model and −6.1 (−9.8, −2.4) in the multivariable model.

e-Table 8. Joint model fits for SF-12 PCS score trajectory.

|  | **Univariable model*** | | | | **Multivariable model*** | | | |
| --- | --- | --- | --- | --- | --- | --- | --- | --- |
| **Trajectory** | **Trajectory intercept**  **(95% CI)** | **P- value** | **Trajectory slope (95% CI)^†^** | **P-value** | **Trajectory intercept (95% CI)** | **P-value** | **Trajectory slope (95% CI)^†^** | **P-Value** |
| Time | 38.8 (38.2, 39.4) | <0.001 | −1.8 (−2.1, −1.5) | <0.001 | 34.2 (28.9, 39.5) | <0.001 | −2.8 (−3.8, −1.8) | <0.001 |
| **Covariates** | **Trajectory intercept**  **difference (95% CI)** | **P- value** | **Trajectory slope**  **difference (95% CI)** | **P-value^‡^** | **Trajectory intercept**  **difference (95% CI)** | **P-value** | **Trajectory slope**  **difference (95% CI)** | **P-value^‡^** |
| Age (per 10 years higher) | −0.4 (−1.2, 0.4) | 0.306 | --- | --- | −0.6 (−1.3, 0.1) | 0.076 | --- | --- |
| Female vs male sex | −0.8 (−2.1, 0.6) | 0.256 | --- | --- | −1.5 (−2.7, −0.3) | 0.011 | --- | --- |
| FVC % predicted (per 10 units higher) | 1.7 (1.4, 2.1) | <0.001 | --- | --- | 1.1 (0.8, 1.4) | <0.001 | --- | --- |
| DLco % predicted (per 10 units higher) | 2.4 (2.0, 2.7) | <0.001 | 0.3 (0.1, 0.5) | 0.005 | 1.1 (0.7, 1.6) | <0.001 | 0.3 (0.1, 0.5) | 0.002 |
| Supplemental oxygen use |  | <0.001 | --- | --- |  | <0.001 | --- | --- |
| At rest vs none^§^ | −8.9 (−10.3, −7.4) |  | --- | --- | −6.3 (−7.8, −4.9) |  | --- | --- |
| With activity only vs none | −5.7 (−7.3, −4.2) |  | --- | --- | −4.1 (−5.6, −2.6) |  | --- | --- |
| Antifibrotic drug use | −1.0 (−2.2, 0.2) | 0.095 | --- | --- | −0.5 (−1.5, 0.5) | 0.348 | --- | --- |

*Univariable models included only time or only time and covariate in longitudinal component of joint model; multivariable model included time and all covariates in longitudinal component.
^†^Trajectory slope estimate reported because there was no evidence of a non-linear relationship with time (p>0.99); trajectory slope estimates reported per 12 months change in HRQL.
^‡^Covariate-time interaction p-value (if applicable).
^§^Included all subjects using oxygen at rest, regardless of whether they used oxygen with activity.

Net change over 48 months (95% CI) was −7.0 (−8.2, −5.8) in the univariable model and −11.3 (−15.3, −7.2) in the multivariable model.

e-Table 9. Joint model fits for EuroQol score trajectory.

|  | **Univariable model*** | | | | **Multivariable model*** | | | |
| --- | --- | --- | --- | --- | --- | --- | --- | --- |
| **Trajectory** | **Trajectory intercept**  **(95% CI)** | **P- value** | **Trajectory slope (95% CI)^†^** | **P-value** | **Trajectory intercept (95% CI)** | **P-value** | **Trajectory slope (95% CI)^†^** | **P-Value** |
| Time | 0.79 (0.77, 0.80) | <0.001 | −0.02 (−0.03, −0.01) | <0.001 | 0.67 (0.55, 0.78) | <0.001 | −0.05 (−0.07, −0.03) | <0.001 |
| **Covariates** | **Trajectory intercept**  **difference (95% CI)** | **P- value** | **Trajectory slope**  **difference (95% CI)** | **P-value^‡^** | **Trajectory intercept**  **difference (95% CI)** | **P-value** | **Trajectory slope**  **difference (95% CI)** | **P-value^‡^** |
| Age (per 10 years higher) | 0.01 (−0.01, 0.02) | 0.364 | --- | --- | 0.01 (−0.01, 0.02) | 0.301 | --- | --- |
| Female vs male sex | −0.03 (−0.06, −0.00) | 0.029 | --- | --- | −0.03 (−0.06, −0.01) | 0.008 | --- | --- |
| FVC % predicted (per 10 units higher) | 0.02 (0.01, 0.03) | <0.001 | --- | --- | 0.01 (0.00, 0.02) | 0.004 | --- | --- |
| DLco % predicted (per 10 units higher) | 0.02 (0.01, 0.03) | <0.001 | 0.01 (0.00, 0.01) | 0.008 | 0.00 (−0.01, 0.01) | 0.449 | 0.01 (0.00, 0.01) | 0.003 |
| Supplemental oxygen use |  | <0.001 | --- | --- |  | <0.001 | --- | --- |
| At rest vs none^§^ | −0.14 (−0.17, −0.11) |  | --- | --- | −0.12 (−0.15, −0.09) |  | --- | --- |
| With activity only vs none | −0.07 (−0.10, −0.03) |  | --- | --- | −0.05 (−0.09, −0.02) |  | --- | --- |
| Antifibrotic drug use | 0.01 (−0.01, 0.04) | 0.351 | --- | --- | 0.02 (−0.00, 0.04) | 0.081 | --- | --- |

*Univariable models included only time or only time and covariate in longitudinal component of joint model; multivariable model included time and all covariates in longitudinal component.
^†^Trajectory slope estimate reported because there was no evidence of a non-linear relationship with time (p>0.99); trajectory slope estimates reported per 12 months change in HRQL.
^‡^Covariate-time interaction p-value (if applicable).
^§^Included all subjects using oxygen at rest, regardless of whether they used oxygen with activity.

Net change over 48 months (95% CI) was −0.08 (−0.11, −0.06) in the univariable model and −0.21 (−0.30, −0.13) in the multivariable model.

e-Table 10. Joint model fits for EuroQol VAS score trajectory.

|  | **Univariable model*** | | | | **Multivariable model*** | | | |
| --- | --- | --- | --- | --- | --- | --- | --- | --- |
| **Trajectory** | **Trajectory intercept**  **(95% CI)** | **P- value** | **Trajectory slope (95% CI)^†^** | **P-value** | **Trajectory intercept (95% CI)** | **P-value** | **Trajectory slope (95% CI)^†^** | **P-Value** |
| Time | 72.1 (71.0, 73.2) | <0.001 | −2.2 (−2.8, −1.7) | <0.001 | 55.4 (45.7, 65.2) | <0.001 | −4.8 (−6.9, −2.7) | <0.001 |
| **Covariates** | **Trajectory intercept**  **difference (95% CI)** | **P- value** | **Trajectory slope**  **difference (95% CI)** | **P-value^‡^** | **Trajectory intercept**  **difference (95% CI)** | **P-value** | **Trajectory slope**  **difference (95% CI)** | **P-value^‡^** |
| Age (per 10 years higher) | 0.9 (−0.4, 2.3) | 0.188 | --- | --- | 0.5 (−0.7, 1.8) | 0.408 | --- | --- |
| Female vs male sex | 1.0 (−1.3, 3.4) | 0.391 | --- | --- | 0.0 (−2.2, 2.2) | 0.987 | --- | --- |
| FVC % predicted (per 10 units higher) | 2.8 (2.3, 3.4) | <0.001 | --- | --- | 1.8 (1.2, 2.4) | <0.001 | --- | --- |
| DLco % predicted (per 10 units higher) | 2.8 (2.1, 3.6) | <0.001 | 0.3 (−0.1, 0.8) | 0.100 | 0.7 (−0.1, 1.5) | 0.093 | 0.5 (0.1, 0.9) | 0.024 |
| Supplemental oxygen use |  | <0.001 |  | 0.019 |  | <0.001 |  | 0.023 |
| At rest vs none^§^ | −14.7 (−17.5, −11.9) |  | 2.1 (0.2, 3.9) |  | −11.7 (−14.6, −8.7) |  | 2.1 (0.3, 4.0) |  |
| With activity only vs none | −7.3 (−10.3, −4.3) |  | −1.2 (−2.7, 0.4) |  | −5.2 (−8.2, −2.2) |  | −0.9 (−2.5, 0.6) |  |
| Antifibrotic drug use | −1.2 (−3.3, 0.9) | 0.250 | --- | --- | −0.1 (−2.0, 1.8) | 0.926 | --- | --- |

*Univariable models included only time or only time and covariate in longitudinal component of joint model; multivariable model included time and all covariates in longitudinal component.
^†^Trajectory slope estimate reported because there was no evidence of a non-linear relationship with time (p>0.99); trajectory slope estimates reported per 12 months change in HRQL.
^‡^Covariate-time interaction p-value (if applicable).
^§^Included all subjects using oxygen at rest, regardless of whether they used oxygen with activity.

Net change over 48 months (95% CI) was −9.0 (−11.3, −6.7) in the univariable model and −19.3 (−27.6, −10.9) in the multivariable model.
